# Pleuropneumonia and mediastinitis caused by *Rhodococcus equi* in a dog: a case report

**DOI:** 10.3389/fvets.2026.1912379

**Published:** 2026-09-22

**Authors:** Tatyana Salarolli, Taismara Simas de Oliveira, Fernanda Caroline Brito Ferreira, Isabela Pádua Zanon, Isabela De Paula Prates, Alex Junio Aparecido da Costa, Tarcísio Resende Júnior, Thaynara Parente de Carvalho, Rodrigo Otávio Silveira Silva, Renato Lima Santos

**Affiliations:** 1 Departamento de Clínica e Cirurgia Veterinárias, Escola de Veterinária, Universidade Federal de Minas Gerais, Belo Horizonte, Brazil; 2 Laboratório Zoogene Ltda. Belo Horizonte, Brazil; 3 Centro de Controle de Zoonoses de Sete Lagoas, Sete Lagoas, Brazil; 4 Departamento de Medicina Veterinária Preventiva, Escola de Veterinária, Universidade Federal de Minas Gerais, Belo Horizonte, Brazil

**Keywords:** MALDI-TOF, mediastinitis, pleuritis, pVAPN, *Rhodococcus equi*, zoonosis

## Abstract

*Rhodococcus equi* is a cosmopolitan bacterium found in the feces of several animals, and it is recognized as a major cause of bronchopneumonia in foals, as well as an opportunistic pathogen causing infections in humans and, rarely, in other species. This report describes a rare and atypical infection by *Rhodococcus equi* in a mixed-breed dog, associated with a pyogranulomatous pleuropneumonia and mediastinitis with abundant intralesional Gram-positive coccobacilli. The bacterium was isolated and identified as *Rhodococcus hoagii* (*R. equi*) using matrix-assisted laser desorption ionization–time of flight (MALDI-ToF), followed by whole-genomic sequencing that detected the *vapN* virulence-associated gene and identified a new sequence type (ST), designated as ST111.

## 1 Introduction

*Rhodococcus equi* is a Gram-positive coccobacillus found in contaminated environments or in the secretions of sick or healthy animals (1). Generally, *R. equi* causes disease in foals, particularly respiratory and intestinal disease (2). The main route of infection is oronasal exposure to a contaminated environment, particularly environments contaminated with equine feces. Although *R. equi* cannot form spores (2), it has high resistance to environmental conditions (2). *R. equi* rarely infects other hosts; however, it has been reported in pigs (3), cattle (4), humans (5, 6), dogs (7), and cats (7), with infection being mostly associated with immunosuppression (8).

It has been suggested that virulence plasmids play an important role in determining the host tropism of *R. equi*. In equine isolates, the gene encoding the virulence-associated protein A (*vapA*) is the most commonly detected virulence gene, whereas *vapB* is more frequently associated with isolates from pigs and wild boars (9). Early studies on rhodococcosis in companion animals failed to identify these virulence factors (7). However, in a study describing five dogs with *R. equi* infections causing endocarditis, endophthalmitis, and suppurative pleuropneumonia, one isolate was found to harbor *vapN*, a novel virulence gene that was initially considered to be associated with ruminant hosts (8). Since then, *vapN*-positive *R. equi* isolates have been reported in humans in several studies, suggesting that this virulence plasmid may have a broader host range than previously recognized (6, 10, 11).

This report describes clinical, pathological, microbiological, and molecular findings of a case of mediastinitis and chronic pleuropneumonia caused by *R. equi* in a dog.

## 2 Case report

A community male, mixed-breed dog (*Canis lupus familiaris*), approximately 7 years old and weighing 21 kg, was rescued by the Fire Department in May 2024 because of a history of aggressive behavior, including attacks on other animals and people, and was sent to the Center for Zoonoses Control (CCZ) in the city of Sete Lagoas (Minas Gerais, Brazil), where the dog underwent orchiectomy. In August 2025, while still housed at the CCZ, the dog developed lethargy, pale mucous membranes, and flea and tick infestations. Hematological examination indicated normochromic normocytic anemia, thrombocytopenia, leukopenia, and hyperglobulinemia, with a presumptive diagnosis of *Ehrlichia canis* infection. The dog was subsequently treated with Imizol (imidocarb dipropionate; 6.6 mg/kg administered subcutaneously in two applications 14 days apart) in combination with doxycycline (200 mg orally once a day for 28 days).

The clinical condition of the dog improved. However, on 8 September 2025, weight loss became clearly visible, and on 15 September, the dog developed severe dyspnea and became lethargic. Emergency interventions were performed, including oxygen therapy, antibiotic therapy with amoxicillin/clavulanic acid (Agemoxi; 15 mg/kg twice a day for 4 days), and analgesia with morphine (0.2 mg/kg twice a day for 2 days). Thoracic radiograph imaging indicated persistent pleural effusion (Figures 1A,B); thus, bilateral thoracentesis was performed to drain approximately 1.8 L of cloudy and reddish fluid. On 17 September, the dog presented with paradoxical breathing, pale mucous membranes, capillary refill time of more than 2 s, and a markedly diminished respiratory sound on auscultation. Despite receiving intensive care, the condition remained refractory to therapy, and on 18 September, the animal developed diarrhea. Due to the poor prognosis, euthanasia was elected, and the dog was immediately submitted for necropsy.

**Figure 1:**
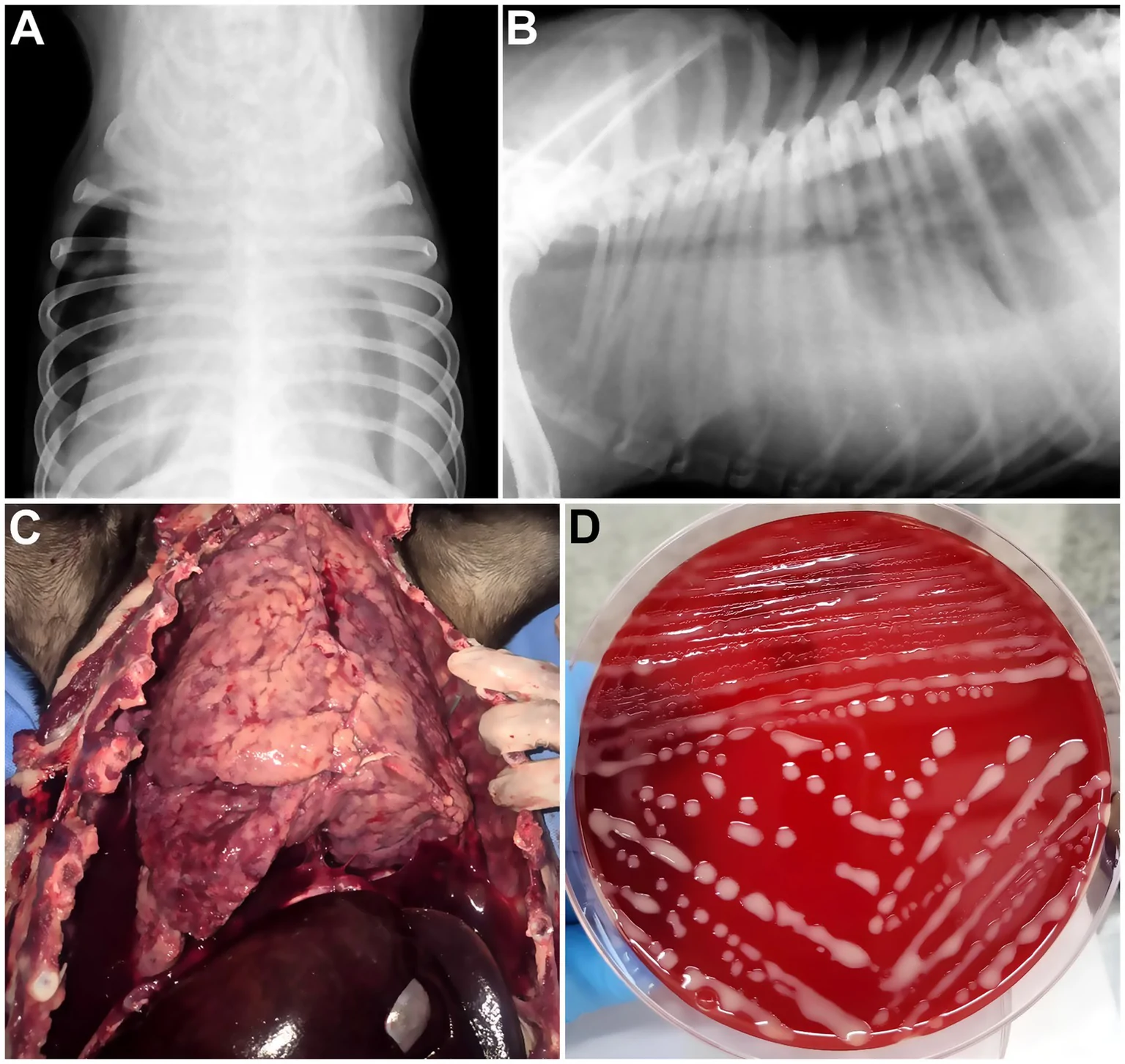
**(A)** Ventrodorsal radiographic image, thoracic cavity with areas of increased radiopacity due to increased volume in the mediastinal region. **(B)** Laterolateral radiographic image, thoracic cavity with areas of increased radiopacity. **(C)** Exposed thoracic cavity, markedly expanded mediastinum with a multinodular mass. **(D)** Blood agar plate with growth of a single bacterial culture of white coloration and milky appearance.

## 3 Diagnostic assessment

### 3.1 Gross pathology

Grossly, there was abundant fluid accumulation in the thoracic cavity, with a thick yellow fibrillar material adhering to the visceral and parietal pleurae, including the thoracic and diaphragmatic pleurae. The mediastinum was diffusely expanded, with a multinodular appearance, and it was soft and white, occupying the entire ventral thoracic region and compressing the adjacent lung parenchyma (Figure 1C). The lung contained multiple nodular consolidated areas, similar to those observed in the mediastinum (Supplementary File 1). Cytological evaluation demonstrated numerous macrophages and neutrophils. Tissue samples were fixed in 10% buffered formalin for histopathology, and swabs were obtained from the mediastinal region, lung, liver, spleen, heart, and intestinal contents. The swabs were refrigerated and sent for bacterial culture (Figure 1D).

### 3.2 Histopathology

Histologically, in the visceral pleura extending into the lung parenchyma, there was a diffuse and severe inflammatory infiltrate consisting predominantly of epithelioid macrophages, with occasional well-defined and large intracytoplasmic vacuoles containing Gram-positive coccobacilli measuring 1–2 μm, which were also observed extracellularly, as well as intracytoplasmically in macrophages without a defined vacuole. There were also aggregates of neutrophils, as well as lymphocytes, plasma cells, and occasional multinucleated giant cells, with multifocal to coalescing areas of fibroplasia and angioplasia. These changes were interpreted as pyogranulomatous pleuropneumonia with intralesional Gram-positive coccobacilli (Figure 2). In the pulmonary parenchyma, there were multifocal areas of alveolar septa thickened by a moderate lymphohistiocytic infiltrate, interpreted as moderate multifocal lymphohistiocytic interstitial pneumonia, with medium-caliber artery thrombosis, moderate diffuse congestion, hemorrhage, multifocal atelectasis, and mild multifocal anthracosis.

**Figure 2:**
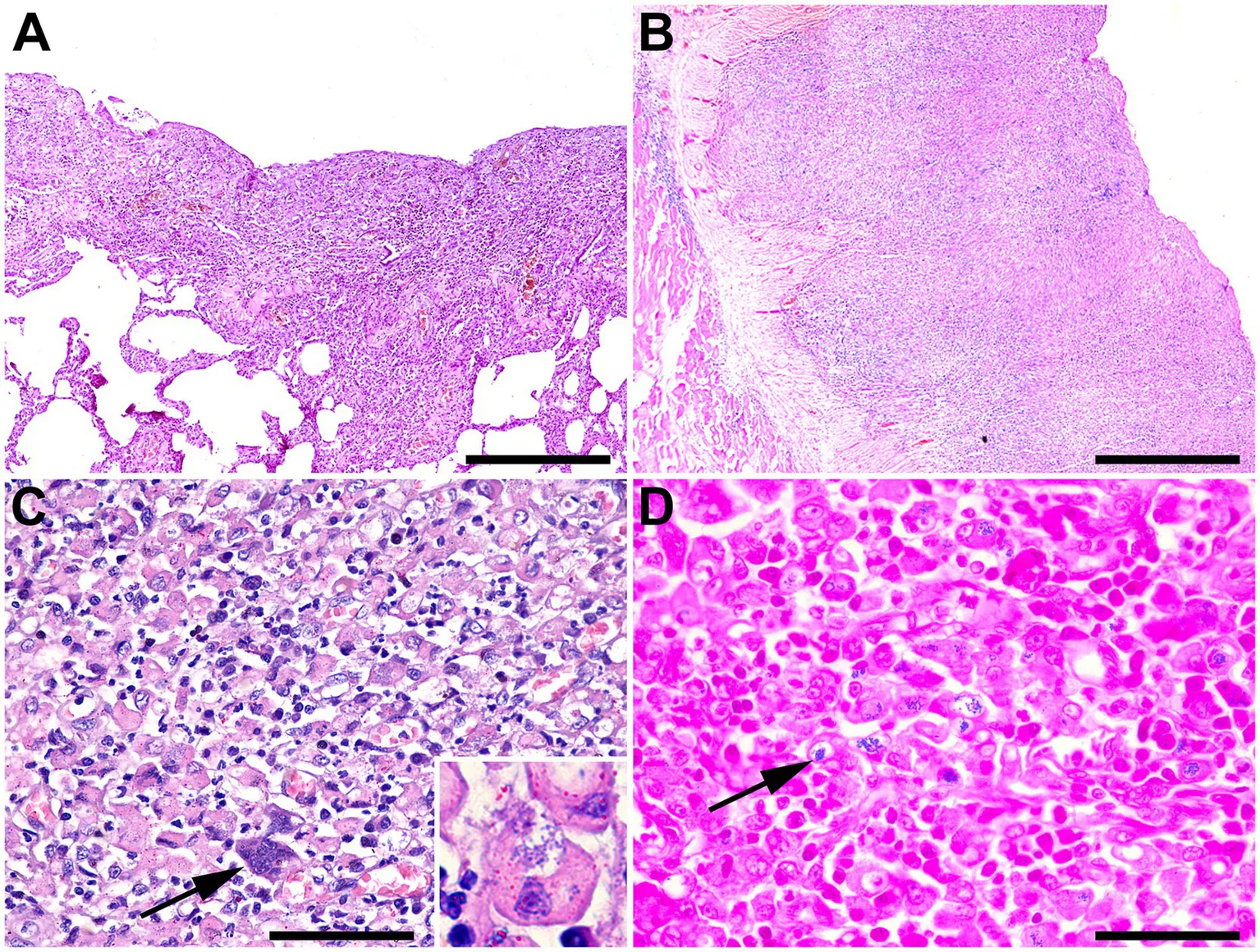
**(A)** Lung, markedly and diffusely expanded visceral pleura with hypercellularity. Hematoxylin–eosin staining, scale bar = 400 μm. **(B)** Diaphragm, parietal pleura of the thoracic surface with marked pyogranulomatous serositis. Hematoxylin–eosin stain, scale bar = 800 μm. **(C)** Mediastinum, pyogranulomatous mediastinitis with numerous epithelioid macrophages and neutrophils, and occasional multinucleated giant cells (arrow); Inset: epithelioid macrophage containing intracytoplasmic coccobacilli. Hematoxylin–eosin staining, scale bar = 100 μm. **(D)** Mediastinum, pyogranulomatous mediastinitis with intralesional Gram-positive coccobacilli (arrow). Gram stain, scale bar = 60 μm.

The multinodular mediastinal mass contained adipose tissue and lymph nodes, with a marked predominantly granulomatous inflammatory infiltrate and bacteria with morphological features similar to those described in the lung, which was interpreted as pyogranulomatous mediastinitis and lymphadenitis. In addition, the thoracic surface of the diaphragm had mesothelial hypertrophy, with diffuse pyogranulomatous serositis and superficial myositis, with features similar to those described in the lung. In the thoracic diaphragm, there was also mild muscle degeneration, with mineralization and necrosis, occasionally associated with hemorrhage and fibrin deposition (Figure 2).

### 3.3 Microbiological isolation and identification

The swabs were plated onto Brain Heart Infusion agar (BHI, Oxoid, UK) supplemented with 5% equine blood and onto MacConkey agar (Kasvi, Italy) and incubated for 48 h under aerobic conditions. From all samples, colonies with typical *Rhodococcus* spp. morphology were cultured on blood agar and characterized as non-hemolytic, opaque colonies with a mucoid to glistening appearance (Figure 1D). Gram staining revealed Gram-positive coccobacilli arranged singly, in pairs, or in short chains. Species identification was performed by matrix-assisted laser desorption ionization–time of flight (MALDI-ToF, Biotyper Compass version 4.1.100) mass spectrometry using the BDAL library V13.0.0.2 (Bruker Daltonics), as previously described (12). The isolate was identified as *Rhodococcus hoagii*, with a score of 2.100. Although *R. hoagii* was rejected under ICSP Judicial Opinion 106, *R. equi* is the currently recommended name (13).

For whole-genomic sequencing, DNA was extracted using the Wizard Genomic DNA Purification kit (Promega, Madison, Wisconsin, USA). Sequencing was then performed on the Illumina MiSeq platform (average output cycles of 2 × 150 bp), and the raw reads were analyzed using FastQC (Babraham Bioinformatics, Cambridge, UK), retaining only paired reads with a Phred quality score ≥30 and a minimum length of 50 nucleotides. Genome assembly was performed using SPAdes 3.5.0 in the careful mode (14), followed by gap filling and polishing with Pilon (15). The isolate was successfully sequenced, achieving an average coverage depth of 69.8x with no evidence of contamination. The genome has been deposited in the NCBI database and is available online under the GenBank accession number JBYVDX000000000 within BioProject PRJNA1390188.

To confirm the identity of the isolate, bacterial species prediction was performed using a K-mer-based algorithm (16), which identified *R. equi* as the likely species, corroborating the results obtained by MALDI-ToF. Then, an *in silico* polymerase chain reaction (PCR) was performed using previously described primers specific for *R. equi* (17), resulting in the expected 450 bp amplicon. Once the species was confirmed, the sequence type was determined using PubMLST (18). The isolate was identified as a new sequence type (ST), and a new ST number was assigned (ST111).

To investigate the presence of virulence genes commonly associated with *R. equi* infections, contigs were analyzed using the virulence factor database (VFDB) (19) and BLASTn analysis against reference *vap* gene sequences (20). The presence of *vapN* was identified by BLASTn and subsequently confirmed by *in silico* PCR using previously described *vapN*-specific primers (8, 21). No additional virulence-associated genes were detected.

Single-nucleotide polymorphism (SNP) analysis was performed using CSIPhylogeny (22), with a minimum Z-score of 1.96 and a minimum depth at each SNP position of 10×, using ATCC 33701 (accession number GCA_002095045.1) as a reference. For a comparative analysis, all publicly available *R. equi* genomes with a minimum coverage depth of 30x were included, totaling 67 isolates, including *vapN*-positive strains, all retrieved from the Bacterial and Viral Bioinformatics Resource Center (BV-BRC) and from the National Center for Biotechnology Information (NCBI) (Supplementary File 2). Only isolates with available metadata regarding both host and country of isolation were included in this study. The phylogenetic tree was visualized using the iTOL v.6 online tool with midpoint rooting (23). The analysis revealed that the isolate from this study differed by at least 11,000 SNPs from the *R. equi* genomes reported in previous studies (Supplementary File 3) and clustered together with previously described *vapN*-positive isolates from humans (Figure 3).

**Figure 3:**
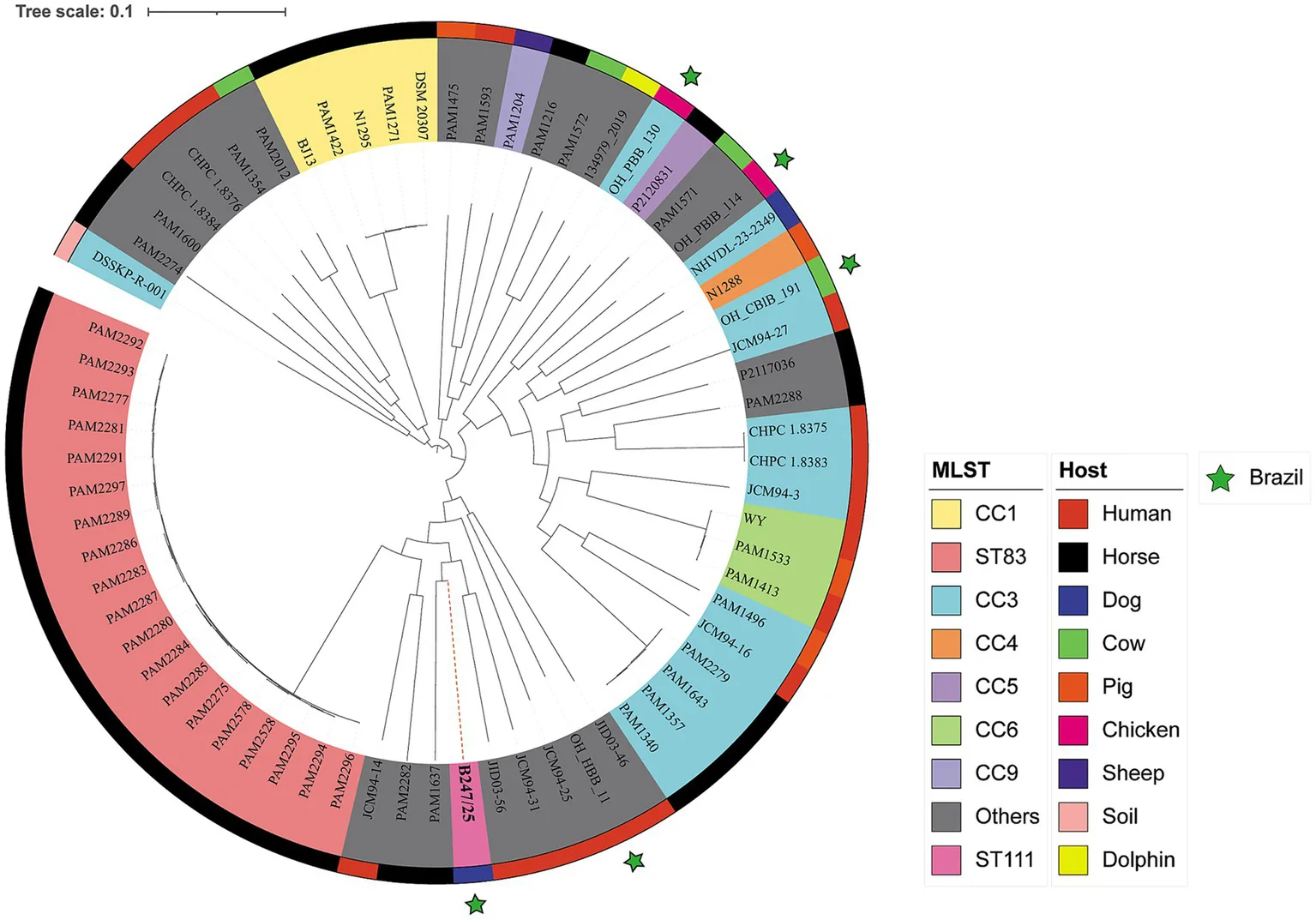
Single-nucleotide polymorphism (SNP) analysis showing the genetic relationship between the canine *Rhodococcus equi* isolate and strains from different hosts, including humans. The tree visualization was generated using iTOL v.6 online (22), applying midpoint rooting.

## 4 Discussion

*Rhodococcus equi* infection is often identified as a cause of bronchopneumonia in foals (2). In dogs, *R. equi*-elicited lesions are uncommon. In this case, the dog developed pleuropneumonia, lymphadenitis, and mediastinitis with intralesional *R. equi*. The most likely, although hypothetical, course of infection in this case involved an initial infection by the pulmonary route, with the development of bronchopneumonia, followed by bacterial phagocytosis by histiocytes, which drained through the lymphatic vessels, disseminating to mediastinal and tracheobronchial lymph nodes, resulting in lymphadenitis (2). Subsequently, the infectious/inflammatory process extended to the mediastinum, followed by the parietal and visceral pleura, culminating in mediastinitis and diffuse pleuropneumonia. Radiographic findings were consistent with pleural effusion and may have obscured chronic lesions that could have been detected radiographically and were later observed at necropsy. Foals with pulmonary rhodococcosis often present with radiographically observable nodules, masses, abscesses, or cavity lesions, although the diagnostic sensitivity and specificity of thoracic radiographs are limited (24).

An exuberant pyogranulomatous inflammation is commonly associated with infection by pathogenic strains carrying virulence-associated plasmids, such as those harboring *vapA*, *vapB*, or *vapN*, which enable intracellular survival by inhibiting phagolysosome fusion within macrophages and consequently preventing bacterial killing (25, 26). In this case, the isolate carried the *vapN* gene, supporting its pathogenic potential and likely contributing to the development of the observed pyogranulomatous lesions (4, 27). The *vapN* gene is primarily associated with bovine isolates (28) but has recently been documented in several human *R. equi* infections. In dogs, *R. equi* infection is considered rare, and only limited information is available regarding the distribution of virulence-associated plasmids (7). Notably, a previous investigation of canine cases identified a *vapN*-positive isolate (8), which, together with our findings, supports the ability of pVAPN-carrying strains to cause infection in dogs.

Coinfections with immunosuppressive pathogens are considered predisposing factors for *R. equi* infection. Therefore, the previous presumptive diagnosis of *Ehrlichia canis* infection, based on clinical examination and therapeutic response, may have played a role as a predisposing factor for *R. equi* infection in this case. *E. canis* is an intracellular bacterium transmitted by the tick *Rhipicephalus sanguineus* that infects monocytes (29). However, the immunosuppressive role of *E. canis* remains controversial (29, 30), and whether *E. canis* may actually predispose to *R. equi* infection remains uncertain. Importantly, *R. equi* itself may induce hematological changes that could have interfered with an accurate assessment of hematologic findings in this case (24). Furthermore, *R. equi* is susceptible to doxycycline (31), which may also have interfered with the interpretation of a positive therapeutic response against a presumptive *E. canis* infection.

Notably, *R. equi* infections are quite challenging to diagnose early, and are associated with high morbidity and mortality, the need for prolonged treatment, and zoonotic potential, thereby posing a significant risk to human health, particularly for animal handlers (32). Importantly, no other dogs or animal handlers at the CCZ reported any health issues that could be linked to this case. *R. equi* poses a risk, particularly to immunosuppressed human patients, among whom a previous history of interactions with animals was frequently reported (33). In addition, many alternative potential host species have been identified (34, 35), which underlines the potential implications of this pathogen from a One Health perspective.

Previous studies have suggested that *R. equi* exhibits host-associated population structures, with certain sequence types being more frequently detected in specific animal species (9, 36). To better understand the genomic context of the isolate described in this study, we performed a SNP-based phylogenetic analysis including *R. equi* isolates from different host species and geographic regions, including human-derived strains. The analysis revealed that the isolate described in this study differed substantially from most deposited *R. equi* genomes. This finding corroborates the results of the multilocus sequence typing analysis, which identified the isolate as belonging to a novel sequence type (ST111). Nevertheless, the isolate clustered with previously described *vapN*-positive strains from a recent study of infected humans (11), suggesting a possible phylogenetic relationship among *vapN*-harboring isolates across different host species.

In conclusion, we report an infection in a dog with a *vapN*-positive *R. equi* strain associated with extensive pyogranulomatous pleuropneumonia and mediastinitis with abundant intralesional coccobacilli and a lethal outcome. The insidious evolution, combined with the atypical manifestation of the disease in an unusual species, delayed a conclusive diagnosis, making it impossible to institute specific and effective antimicrobial therapy. This case underscores the importance of recognizing the zoonotic potential of the agent, since the absence of early diagnosis exposed professionals and community members to an opportunistic pathogen with the potential to cause human disease.

## Data Availability

The datasets presented in this study can be found in online repositories. The names of the repository/repositories and accession number(s) can be found at: https://www.ncbi.nlm.nih.gov/genbank/, JBYVDX000000000.
